# Toxicity of iron oxide nanoparticles to grass litter decomposition in a sandy soil

**DOI:** 10.1038/srep41965

**Published:** 2017-02-03

**Authors:** Muhammad Imtiaz Rashid, Tanvir Shahzad, Muhammad Shahid, Muhammad Imran, Jeyakumar Dhavamani, Iqbal M. I. Ismail, Jalal M. Basahi, Talal Almeelbi

**Affiliations:** 1 Center of Excellence in Environmental Studies, King Abdulaziz University, P. O. Box 80216, Jeddah 21589, Saudi Arabia; 2 Department of Environmental Sciences, COMSATS Institute of Information Technology, 61100, Vehari, Pakistan; 3 Department of Environmental Sciences & Engineering, Government College University, 38000, Faisalabad, Pakistan; 4 Department of Chemistry, Faculty of Science, King Abdulaziz University, P. O. Box 80203, Jeddah 21589, Saudi Arabia; 5 Department of Environmental Sciences, King Abdulaziz University, Jeddah 2158, Saudi Arabia

## Abstract

We examined time-dependent effect of iron oxide nanoparticles (IONPs) at a rate of 2000 mg kg^−1^ soil on *Cynodon dactylon* litter (3 g kg^−1^) decomposition in an arid sandy soil. Overall, heterotrophic cultivable bacterial and fungal colonies, and microbial biomass carbon were significantly decreased in litter-amended soil by the application of nanoparticles after 90 and 180 days of incubation. Time dependent effect of nanoparticles was significant for microbial biomass in litter-amended soil where nanoparticles decreased this variable from 27% after 90 days to 49% after 180 days. IONPs decreased CO_2_ emission by 28 and 30% from litter-amended soil after 90 and 180 days, respectively. These observations indicated that time-dependent effect was not significant on grass-litter carbon mineralization efficiency. Alternatively, nanoparticles application significantly reduced mineral nitrogen content in litter-amended soil in both time intervals. Therefore, nitrogen mineralization efficiency was decreased to 60% after 180 days compared to that after 90 days in nanoparticles grass-litter amended soil. These effects can be explained by the presence of labile Fe in microbial biomass after 180 days in nanoparticles amendment. Hence, our results suggest that toxicity of IONPs to soil functioning should consider before recommending their use in agro-ecosystems.

Leaf litter decomposition is an essential process for the functioning of natural or agro-ecosystems. This process is a primary step in the nutrient cycling of aforementioned ecosystems, therefore acts as a mediator in providing food for living organisms, building up organic matter^1^ and a source of carbon dioxide emission from soil^2^,^3^. In natural ecosystems like forest, 68–87% demand of the essential nutrients required for annual plant growth can be fulfilled by the process of leaf litter decomposition and mineralization-immobilization turnover of nutrients^4^. Consequently, litter decomposition is a principal process in maintaining the ecosystem stability^5^. This process is mainly commuted by microbial diversity and detritivorous animals in the soil^6^,^7^. Microbes play an important role in nutrient cycling and transfer of energy from leaf litter decomposition to higher trophic levels in the soil food web^8^. Therefore among others, soil microbial decomposers are the most important factors influencing decomposition process at ecosystem level and thereby the ecosystem services^7^,^9^. On the other hand, soil is under continuous stress of environmental disturbances caused by very intensive human practices especially in agricultural ecosystems^10^. Therefore, any turmoil caused due to anthropogenic stressors would influence the activity or diversity of microorganisms in the soil and may indirectly affect soil functionality such as leaf litter decomposition^11^.

In recent years, increasing use of iron oxide nanoparticles (IONPs) for crop protection, fertilization and remediating soil organic pollutants in agriculture^12^,^13^,^14^,^15^,^16^ has been observed. For instance, He *et al*.^17^, observed that IONPs positively affected soil microbial activity and nitrification potential. Similarly, a higher root and shoot phosphorous uptake was observed when IONPs were applied to *Lactuca sativa*^18^. Application of IONPs increased the root and shoot biomass of pumpkin and rye grass^16^ and promoted the growth of tomato^19^. Despite such positive effects of nanoparticles on some of the plant growth parameters, their toxicity on soil living organisms could not be negated while taking into account the soil-plant interactions. For instance, use of iron oxide nanoparticles decreased the microbial activity and nutrient acquisition by fungi in soil^20^,^21^,^22^. Consequently, the metabolic quotient, a measure of soil pollution was higher in IONPs treated compared to control soil, indicating nanoparticles stress to soil microbial activity^20^. Moreover, such nanomaterials would be accumulated or taken up by soil microbes during their life cycle^20^. From there, these nanoparticles would be transferred to biotic predators in the soil food web, and may have direct or indirect consequences on biological or ecological processes or functions in the soil^11^.

A very recent study indicated that zero valent iron nanoparticles decreased the root, shoot length as well as chlorophyll and carotenoids content in rice^23^. This study concluded that nanoparticles damaged the cortex tissue thereby blocking the active transport of iron into rice root and shoot. Similarly, application of IONPs in soil significantly decreased the biomass of clover that was associated with arbuscular mycorrhizal fungi^21^. Such effects could be explained by reduction in the glomalin production capacity and nutrient acquisition by fungi through nanoparticles^21^. Also, bacterial activity and diversity were found to be lower in IONPs treated soil having low organic matter and clay content compared to a clayey soil rich in organic matter^22^. In addition to aforementioned soil parameters, pH, soil salinity and ionic strength also influenced nanoparticles toxicity or bioavailability in the soil^24^,^25^. Such parameters determined the nanoparticles dissolution, agglomeration or aggregation in soil solution and thus their stability in soil^25^. However, stability of IONPs is weak due to higher mobility of electron within their structure and diffusion of Fe^2+^ ions^26^. This effect would influence its bioavailability in the soil with time and hence its toxicity. Consequently, studies regarding the nanoparticles behavior in soil are required over various incubation times on a scale of months to years^20^,^25^.

Soil microbiota are the major drivers of litter decomposition and nutrient cycling in an agroecosystem^7^,^27^,^28^,^29^ but only a single, very recent study reported the influence of IONPs on soil microbial community and nitrification process when applied at very low doses (0.1–10 mg kg^−1^ soil) in a short incubation interval of 48 hrs^17^. Thus, a very little evidence exists in the literature about the effect of IONPs on microbial communities and their associated functions in the soil. Moreover, most of the studies regarding the impact of IONPs on soil microbes or their associated functions were conducted in culture media under artificial conditions in a very short time interval^17^,^20^,^21^,^22^. Therefore, their applicability to microbial-associated responses and functions in agro-ecosystems is undefined. Equally, their interaction effects with in the soil food web and on ecosystem processes such as litter decomposition, and carbon and nitrogen mineralization from leaf litter are largely unknown.

The objective of the current study was to investigate the time-dependent effect of IONPs on soil microbial biomass, microbial colony forming units, and carbon and nitrogen mineralization of grass litter in mesocosms containing sandy soil over a relatively longer incubation durations (90 and 180 days). We hypothesized that application of IONPs will decrease the microbial biomass and activity in the sandy soil. This decrease in microbial activity would result in lower CO_2_ emission, dissolved organic carbon and mineral nitrogen availability from applied grass-litter in this soil. We also hypothesized that IONPs stability will decrease with time resulting in overall decrease in their toxicity to litter carbon and nitrogen mineralization in the soil.

## Results

### IONPs effect on soil chemical properties

Neither the application of nanoparticles nor grass litter influenced soil organic matter, organic carbon and total nitrogen after 90 and 180 days of incubation intervals (P > 0.05; Table 1). In contrast, application of grass litter significantly decreased the soil pH after 90 days compared to control soil (CS) (P = 0.000). However, it slightly increased after 180 days of soil incubation in the aforementioned treatments (P = 0.003). Nevertheless, treatment and time interaction for this parameter was not significant (P > 0.05). On the other hand, electrical conductivity in both grass litter-amended soil (LS) and nanoparticles-grass litter amended soil (LNPS) was 42% and 49% higher, respectively compared to CS treatment after 90 days of incubation (P = 0.000) but after 180 days this difference decreased to 38% in both treatments (Table 1). However, effect of time on this parameter was not significant (P > 0.05).

### IONPs effect on soil microbial properties

**Figure 1:**
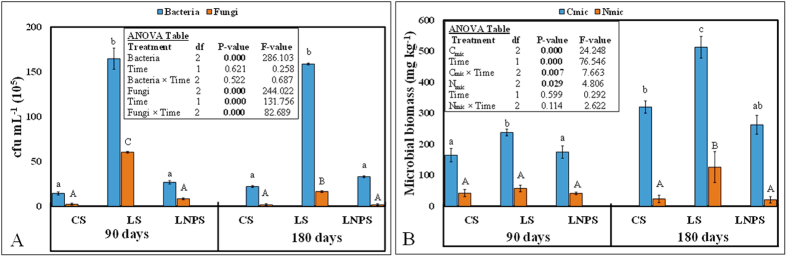
Mean (n = 3) of heterotrophic cultivable colony forming units (10^5^ cfu mL^−1^) of bacteria and fungi (**A**) and microbial biomass carbon (C_mic_) and nitrogen (N_mic_) (**B**) in control soil without any amendment (CS) as well as soil amended with grass litter (LS), and grass litter-nanoparticles (LNPS) after 90 and 180 days of incubation. Bars on panel denote the standard error (±1) of mean. Small letters indicate the significant differences among treatments and time for Bacteria as well as for C_mic_, whereas capital letters indicate this difference for Fungi and N_mic_, at probability level of 5%.

Bacterial colony forming units (*cfu*) were 84% lower in LNPS compared to LS treatment after 90 days of incubation (P = 0.000), however this difference decreased to 79% after 180 days (Fig. 1A). Effect of time on bacterial *cfu* was not significant (P > 0.05). In case of heterotrophic viable fungal counts, IONPs decreased the *cfu* to 83 and 89% after 90 and 180 days of litter incubation, respectively (P = 0.000). Additionally, fungal *cfu* strongly decreased with time (P = 0.000). After 180 days, this decrease was 80% for LNPS (P = 0.000) and 72% for LS treatment (P = 0.011) compared to 90 days of incubation however; no decrease of fungal *cfu* with time in control soil was observed (Fig. 1A).

Microbial biomass carbon (C_mic_) and nitrogen (N_mic_) was also affected by the application of IONPs in grass litter-amended soil (Fig. 1B). For instance, C_mic_ in LNPS treatment was 27% lower than LS treatment after 90 days and this difference increase to 49% after 180 days of incubation (Fig. 1B). Similarly, time also influenced this parameter within each treatment. After 180 days of litter incubation, C_mic_ was increased by 115%, 94% and 50%, in LS, CS and LNPS treatments (P < 0.05), respectively compared to this parameter after 90 days of incubation. Hence, the lowest increase in C_mic_ was observed in LNPS treatment. In case of N_mic_, treatment effect was not significant after 90 days of incubation however, N_mic_ was 83% lower in LNPS compared to LS treatment (P = 0.045). Moreover, no difference in N_mic_ was observed between CS and LNPS treatments (P > 0.05). Time duration did not affect the N_mic_ in CS, LS or LNPS treatments (P > 0.05).

### Grass litter decomposition and nitrogen mineralization

**Figure 2:**
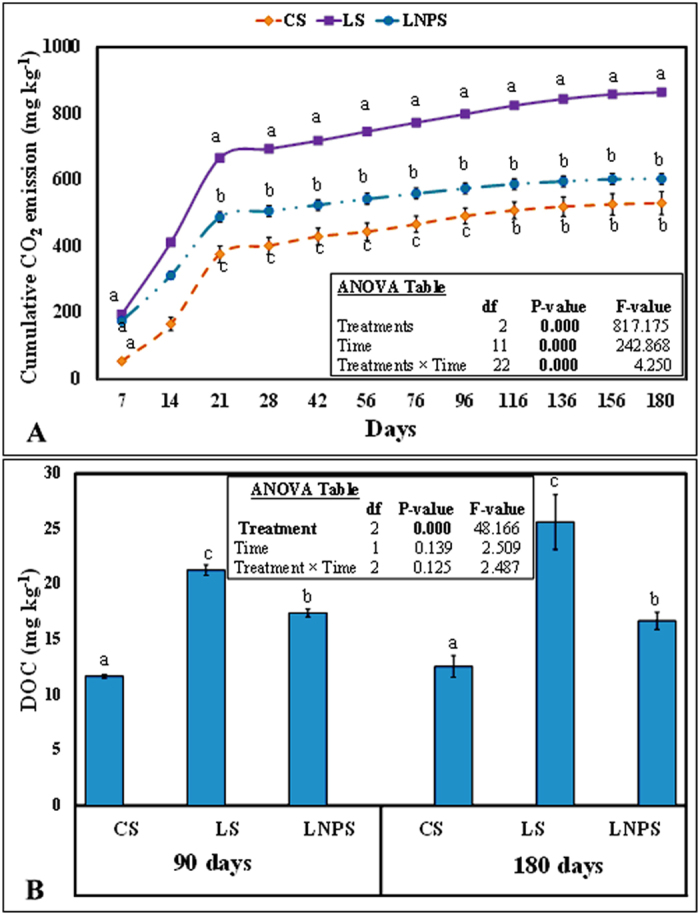
Cumulative CO_2_ efflux (mg CO_2_ kg^−1^ soil; (**A**) and dissolved organic carbon (DOC; (**B**) in control (CS) grass litter-amended (LS), and grass litter-iron oxide nanoparticles amended soil (LNPS). Values are means of three replicates (n = 3) with error bars (±1) represent standard error of the mean. Significant difference among treatments and time represented by small letter at 5% probability level.

Decomposition of grass litter was measured by CO_2_ emission from the treatments as well as dissolved organic carbon present in the soil (Fig. 2A,B). Overall, application of IONPs in grass litter-amended soil decreased the cumulative CO_2_ emission by 30% compared to litter-amended soil after 180 days of incubation (P = 0.000; Fig. 2A). Besides, cumulative CO_2_ emission from LNPS treatment was 14% higher than CS treatment; however, this difference was not significant (P > 0.05). Additionally, cumulative CO_2_ emission from all treatments was significantly affected by time, and interaction between time and treatment was also significant (P = 0.000; Fig. 2A). For instance, cumulative CO_2_ emission linearly increased until 21 days of incubation (P = 0.000) in each treatment but this emission was slowed down afterwards. The reduction in cumulative CO_2_ emission was higher in CS and LNPS treatments compared to LS (Fig. 2A). Effect of treatments on DOC in soil almost followed the same trend as of CO_2_ emission but not the time. This parameter was 18 and 35% lower in LNPS as compared to LS treatment after 90 and 180 days of incubation, respectively (Fig. 2B). However, DOC was not significantly different between CS and LNPS treatment after 90 or 180 days of incubation (P > 0.05; Fig. 2B). On the other hand, IONPs application significantly decreased carbon mineralization efficiency of grass litter. The efficiency in LNPS treatment was 178% lower than LS after 180 days of incubation (P = 0.000, Table 1). This mineralization efficiency was not significantly affected by the incubation duration (P > 0.05; Table 1).

**Figure 3:**
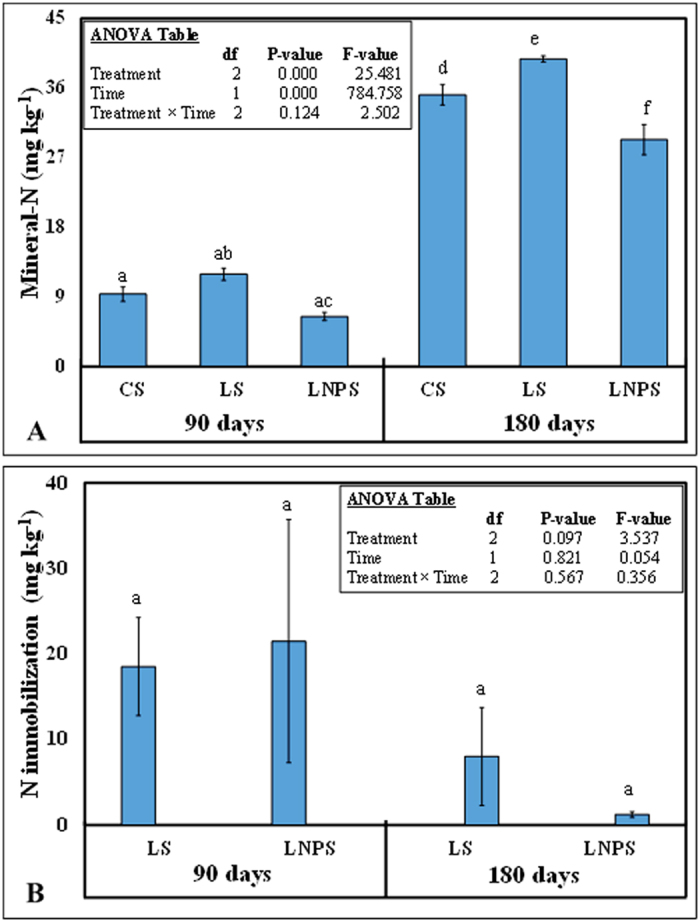
Mineral nitrogen (**A**) and nitrogen immobilization (**B**) in control soil (CS) as well as soil amended with grass litter (LS), and grass litter-nanoparticles (LNPS) after 90 and 180 days of incubation. Error bars represent the standard error (±1) of the mean (n = 3). Significant difference among treatments and time represented by small letter at 5% probability level.

Mineral nitrogen was significantly affected by both treatments and time (P = 0.000; Fig. 3A). Among treatments, IONPs application in litter-amended soil decreased mineral N by 47 and 26% compared to litter-amended soil after 90 and 180 days of incubation, respectively (P < 0.05). However, mineral nitrogen in CS was not significantly different from LS or LNPS treatments after 90 days of incubation. After 180 days, mineral nitrogen content in CS and LS treatments was significantly higher compared to LNPS treatment (Fig. 3A; P < 0.05). This parameter significantly increased with time and was ~3× higher in all treatments after 180 days compared to 90 days of incubation (P < 0.05). Such effects were also observed on nitrogen mineralization efficiency that was significantly lower in LNPS compared to LS treatment (Table 1). For instance, nitrogen mineralization efficiency was 161% lower in LNPS compared to LS treatment after 90 days but this difference increased to 189% after 180 days of incubation study (P = 0.05; Table 1). Contrary to the mineral nitrogen, immobilization of this element in soil was not significantly affected by either treatment or time (P > 0.05; Fig. 3B).

**Figure 4:**
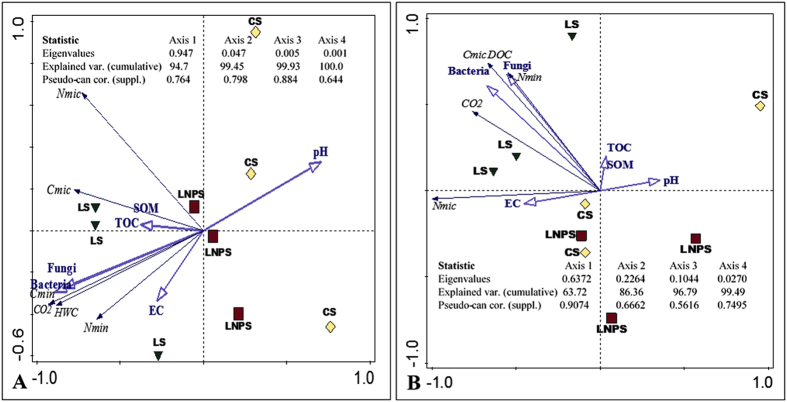
Principal component analysis (PCA) of various soil parameters after 90 (**A**) and 180 days (**B**) of incubation; organic matter (SOM), total organic carbon (TOC), electrical conductivity (EC), pH, bacterial and fungal colony forming units, microbial biomass carbon (C_mic_), nitrogen (N_mic_), dissolved organic carbon (DOC) carbon dioxide (CO_2_) emission, and nitrogen (N_min_) mineralization from grass litter in different treatments. Control soil represented by diamonds (CS), grass litter amended soil in triangles (LS) and grass litter, nanoparticles-amended soil (LNPS) in square. Inset shows the statistics of the analysis where most of the variations in the data are explained by PC1 and PC2, and the individual scores are unit less.

The PCA analysis revealed that most of the data in all tested parameters is explained by first two canonical axes, 86% after 90 days and 99% after 180 days of incubation study. It is apparent from both incubation intervals that IONPs application significantly affected all parameters in litter-amended soil, therefore most of these parameters are in opposite direction to LNPS (Fig. 4A,B). However, after 90 days of incubation, pH was closely associated with LNPS or CS treatments (Fig. 4A). Besides, carbon (CO_2_ emission and DOC) and nitrogen mineralization are closely related to grass litter-amended soil and not with control or LNP-amended soil during both incubation intervals. This was also depicted from length and direction of the arrows (Fig. 4A,B).

### Pollution indices

**Figure 5:**
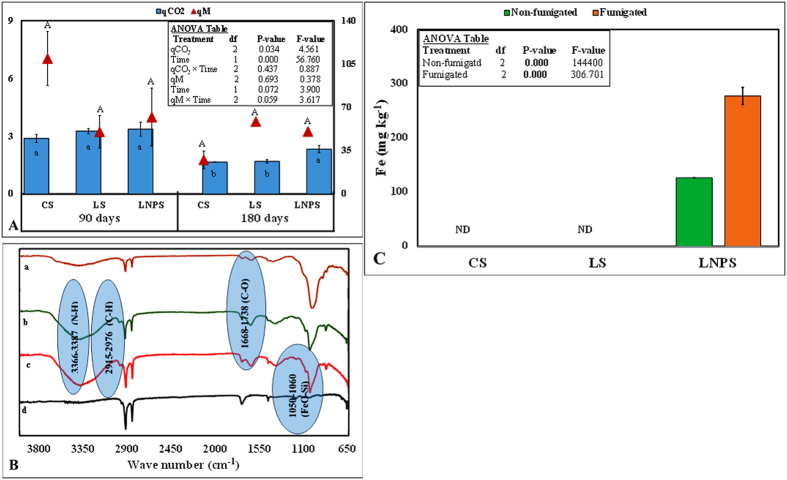
Nanoparticles pollution indices (**A**) in control soil (CS) as well as soil amended with grass litter (LS), and grass litter-nanoparticles (LNPS) after 90 and 180 days of incubation. CO_2_-C emission per unit of microbial biomass carbon (mg C–CO_2_ g^−1^ C_mic_) represented by metabolic quotient (qCO_2_) in the primary axis, and microbial quotient (qM) is expressed as microbial biomass carbon per total organic C (mg kg^−1^) in soil in secondary axis. Small letters in the panel indicate the differences among treatments and time for qCO_2_, and capital letters over error bars show the significant different for qM at 5% probability level. Fourier transform infrared spectra (**B**) in CS (a; dark red) LS (b; green), LNPS after 180 days of incubation (c; red) and iron oxide nanoparticles only (d; black). Microbial biomass Fe in fumigated and non-fumigated soil of CS, LS and LNPS treatment (**C**).

Pollution indices for nanoparticles are presented in Fig. 5A. In general, both treatments and time significantly affected the metabolic quotient (qCO_2_; P < 0.05); however, treatment and time interaction for this parameter was not significant (Fig. 5A). This index was not affected by nanoparticles application in grass litter after 90 days of incubation (P > 0.05), however after 180 days, qCO_2_ was significantly higher in LNPS than LS treatment showing nanoparticles toxicity to litter decomposition (Fig. 5A). On the other hand, microbial quotient (qM) was not affected by treatment or time (P > 0.05) though this parameter tended to be higher in LNPS treatment compared to LS or CS after 180 days of incubation (Fig. 5A).

### Fourier transform infrared (FTIR) spectroscopy of soil

Soil samples from all treatments were subjected to fourier transform infra-red (Shimadzu IRAffinity-1, Kyoto, Japan) spectroscopy. Specifically, 2 mg of each sample was mixed with 200 mg KBr (FTIR grade) to make a pellet. This pellet was placed in sample holder and IR spectra was recorded in ATR mode with 100 scans and 6 cm^−1^ resolutions at a range of 4000–650 cm^−1^. FTIR spectra of all the treatments and IONPs are given in Fig. 5B. These spectra show vibration bands at 3371, 2915, 1668, 1738 and 1050 cm^−1^. The spectral bands at 1050 and 1060 cm^−1^ are assigned to FeO-Si stretching vibrations (spectra c and d, respectively; Fig. 5B). This spectral region is not visible/activated in CS or LS treatments (spectrum a, b; Fig. 5B) showing presence of nanoparticles in the polluted soil. Such effects were also confirmed by the occurrence of high Fe in the microbial biomass in LNPS treatment (Fig. 5C). The spectral bands 1738–1678 cm^−1^ in spectra a, b and c (Fig. 5B) indicate C-O stretching that are safely assigned to carboxylic acid group, a representative of sugar, saccharides, protein and flavonoids. A shift in spectral band from 2915 to 2976 cm^−1^ as well as 3366 to 3387 cm^−1^ (spectrum b, c, respectively; Fig. 5B) shows that nanoparticles may have affected the C-H and N-H stretching vibrations, respectively in the soil. The former stretching is the characteristics of alkanes while the latter for amines group.

## Discussion

In this study, we tested mainly two hypotheses: i) application of IONPs in litter-amended soil will decrease the microbial colony forming units, microbial biomass carbon and nitrogen and microbial activity measured as CO_2_ emission. ii) Consequently, the soil processes of carbon and nitrogen mineralization from grass litter will be modified. As expected, we found that application of nanoparticles decreased the heterotrophic cultivable bacterial and fungal colony counts in grass-litter amended soil (Fig. 1A). The colony counts for both microbial groups were similar in control and LNPS treatments (Fig. 1A) showing that nanoparticles would not allow microorganisms to colonize litter and increase or multiply their biomass in the soil. Such observations are in line with our results of microbial biomass carbon and nitrogen (Fig. 1B) displaying a similar trend as observed in case of microbial colony counts in all treatments. These results are in accordance with Antisari *et al*.^20^ who did not find any effect of IONPs pollution on microbial biomass carbon and nitrogen in a Cambisol. However, they observed higher microbial C:N ratio in soil polluted with nanoparticles and explained this effect by changes in microbial community composition due to nanoparticles toxicity. Moreover, they argued that increase in C:N ratio could probably be due to the dominance of ectomycorrhizae. This argument is supported by Burke *et al*.^30^ who found that arbuscular mycorrhizal root colonization tended to be higher in IONPs contaminated soil, although such colonization was not significantly different between control and nanoparticles amended soil. These explanations could be the plausible reasons for no difference in microbial biomass carbon, nitrogen or microbial colony counts between control, and nanoparticles-litter amended soil in our study.

In accordance with our second hypothesis we found that, carbon mineralization (CO_2_ emission and DOC) was significantly lower in LNPS treatment (Fig. 2A,B). Our findings are in line with Frenk *et al*.^22^ who observed higher decrease in microbial activities and CO_2_ emission in soil with low organic matter content compared to that of soil with high organic matter content after application of IONPs. They explained that low organic matter soils are more vulnerable to anthropogenic stressors due to relatively lower microbial diversity and richness. These parameters are very good predictor of any ecosystem functional stability, including soil prone to disturbances^31^. Organic matter content in soil of our study was quite low (0.29%) that could not decrease the free availability of nanoparticles in the soil (Fig. 5C). In contradiction to Frenk *et al*.^22^ and our study, He *et al*.^17^,^26^, found higher enzymes activities as well as CO_2_ emission in soil amended with high or medium concentration of IONPs compared to their low concentration. They described that higher concentration of IONPs in their case may have caused changes in microbial community composition^26^, though not affecting their abundances, thereby increasing carbon losses through CO_2_ emission. Such phenomenon was not observed in our study, which could be explained by the fact that we used relatively higher dose (2000 mg kg^−1^ soil) of nanoparticles compared to the highest dose (10 mg kg^−1^ soil) used in their study^17^. Therefore, the available amount of Fe from the latter dose may only be used to fulfill the metabolic requirement of microbial community present in such soil^17^ compared to the concentration of nanoparticles used in our study or in case of Ben-Moshe *et al*.^32^, Burke *et al*.^30^, and Frenk *et al*.^22^ PCA analysis revealed that both DOC and CO_2_ are very closely related to fungal and bacterial colony forming units after both incubation intervals and even after 180 days, DOC was strongly correlated with C_mic_ (Fig. 4A,B). These associations are strong indications that IONPs not only decreased the microbial community but their functions of leaf litter decomposition and carbon mineralization. Hence, this decrease in CO_2_ emission and DOC due to nanoparticles application in litter-amended soil caused the lower carbon mineralization efficiency in this treatment compared to litter-amended soil (Table 1).

As expected, IONPs application decreased the nitrogen mineralization from the grass litter applied in sandy soil (Fig. 3A). This was accompanied by lower bacterial and fungal colony forming counts as well as microbial biomass nitrogen in this treatment (Fig. 1A,B). Such observations indicated that IONPs application reduced the microbial activity that in turn lowered the decomposition and nitrogen mineralization from applied litter in soil. According to Feng *et al*.^21^, IONPs reduced the glomalin protein and nutrient acquisition of arbuscular mycorrhizal fungi in the soil although these nanoparticles did not influence AMF infection rate to clover or Fe absorption by this crop. He *et al*.^17^ reported similar effect of IONPs on soil microbial community and found no difference in bacterial community at any concentrations of these nanoparticles, however soil nitrification potential was increased with low and intermediate concentration of nanomaterials but at higher concentration, no difference in soil nitrification potential was observed compared to control soil. In our study, the plausible explanation of the negative effects on grass litter nitrogen mineralization could be that IONPs possess small size and negative zeta potential like biogenic silver nanoparticles^33^ which may allow them to percolate in the microbial cell wall and may eventually disrupt it, hence leading to their death^34^. Consequently, their functions of leaf litter decomposition and nitrogen mineralization may greatly be affected. Such phenomenon could have occurred in our study leading to lower microbial biomass carbon, nitrogen, and tended to lower nitrogen immobilization in nanoparticles, grass litter-amended soil (Figs 1, 2 and 3B). Moreover, this could also be confirmed by PCA analysis after 180 days of incubation in our study, which revealed that mineral nitrogen was closely associated with microbial counts as well as microbial biomass carbon (Fig. 4b). Such effects of nanoparticles resulted in 235% lower nitrogen mineralization efficiency of grass-litter in sandy soil (Table 1).

In contrary to our third hypothesis, we did not find much difference in DOC, and mineral nitrogen over time although overall effect on CO_2_ was significant (P = 0.000). The difference in CO_2_ emission between LNPS and LS treatments was 224 ± 14 mg kg^−1^ after 90 days whereas this was only 261 ± 7 mg kg^−1^ soil after 180 days of incubation. This could be linked to no significant differences observed in microbial colony counts and microbial biomass nitrogen as well as nitrogen immobilization with time in these treatments (Figs 1A and 3B). Although microbial biomass carbon increased significantly CS and LS treatments with time, and the difference for this parameter between LNPS and LS after 180 days (250 ± 7) was significantly higher than after 90 days (63 ± 29) of incubation (P = 0.003). This indicates that IONPs toxicity to microbial life was increasing with time but not with their associated soil functions. In line with this observation, the difference in CO_2_ emission between LS and LNPS treatments tended to be higher after 180 than 90 days of incubation. Moreover, metabolic quotient was only significantly higher after 180 days in LNPS than LS or CS treatments showing stress of nanoparticles to microbial life or their functions of carbon and nitrogen mineralization of grass litter in soil (Fig. 5A). This was also confirmed by the presence of iron in microbial biomass carbon only in LNPS treatment after 180 days of incubation (Fig. 5C); however, iron was not detected in any treatment after 90 days of litter incubation.

## Conclusions

We reported for the first time in a relatively longer time duration study that IONPs significantly decreased the bacterial and fungal colony counts, microbial biomass carbon, and nitrogen as well as grass litter carbon and nitrogen mineralization in a litter-amended sandy soil. IONPs time dependent effect on nitrogen mineralization efficiency was significant indicating that their toxicity significantly reduced the soil function of nutrient mineralization, though such effect was not observed in case of grass-litter carbon mineralization. The former effect was also confirmed by higher metabolic quotient in nanoparticles litter-amended soil. Hence, taking into account that IONPs are toxic to grass litter decomposition, nitrogen mineralization and may affect other ecosystem services, it is recommended to rethink on their use in agriculture for fertilization or remediation purposes.

## Methods

A mesocosm experiment was performed over a period of 180 days in laboratory facility at CEES, King Abdulaziz University, Jeddah, Saudi Arabia. The powdered form of Fe_2_O_3_-NPs was used (Sigma Aldrich, Saint Louis, MO, USA) to assess their toxicity on grass litter decomposition and nitrogen mineralization over the aforementioned time period. The cubical crystal phase nanoparticles had a surface area of 50–245 m^2^ g^−1^ and their size and density were <50 nm and 5.25 g cm^−3^, respectively (manufacturer information). According to Nhan *et al*.^35^, these NPs hydrodynamic size and zeta potential were 154.3 nm and −9.27 mV, respectively. An arid sandy loam soil was collected from the University vicinity. By using a 4 mm mesh screen, we removed root and organic debris from the collected soil. This fresh soil was used to fill the mesocosms, so that natural soil biota community would remain as such in the soil. Grass litter (*Cynodon dactylon*) was collected from the university campus, crushed into ~2 cm, air-dried and stored for further study. Chemical characteristics of grass litter and soil fertility parameters are given in Table 2.

### Experimental design

Mesocosms were prepared from plastic jars^11^ with a surface area of 0.02 m^2^. Each mesocosm was filled with 1 kg of fresh field moist soil. The filled soil covered only half area of the mesocosm, so the rest half remained empty throughout the incubation period. Three treatments, each being replicated thrice, were used i) control soil (CS), ii) grass litter amended soil (LS) and iii) nanoparticles, grass litter amended soil (LNPS). In LS and LNPS treatments, 3 g of the grass litter was thoroughly mixed in the soil. For NPs application in LNPS treatment, typically, it is observed that the concentration of IONPs applied in the soil as a fertilizer, monitoring NPs toxicity or remediating soil pollution ranged between 0.1 to 34000 mg kg^−1^ soil^36^,^37^. Therefore, a nominal realistic concentration of IONPs (2000 mg kg^−1^ soil) in powder form was applied in LNPS treatment^36^,^37^,^38^ and completely mixed in the soil with spatula. Distilled water was periodically showered in all mesocosms to maintain the moisture content (50%) based on weight difference method. The mesocosms were arranged in a completely randomized design inside a dark wooden box that was kept outside where mean temperature during the experimental period was 30.3 °C.

### Carbon dioxide (CO_2_) measurement

A sodium hydroxide (NaOH) trap was used to capture CO_2_ emitted from all treatments^2^,^29^. Specifically, a 10 ml of 1 M NaOH was pipetted in a plastic petri plate and placed inside each mesocosm. All plates were removed and replaced with freshly prepared NaOH at 8 sampling events till 90 days and then 9^th^ event for CO_2_ capture from all the mesocosms was carried out in last two weeks of incubation. Since increase in respiration rate during last four sampling events indicated that CO_2_-production became more or less constant. Therefore, CO_2_ production from all treatments during remaining period of litter incubation was calculated based on CO_2_ production during first incubation period and last two weeks of experiment^39^. After each sampling occasion, mesocosms were tightly sealed with screw lid and self-adhesive tape was overlapped on junction of lid and jar to circumvent escaping of CO_2_ from experimental units. Additionally, ambient concentration of CO_2_ was corrected by placing the petri plates with same amount of NaOH in empty mesocosms (blanks). To quantify the amount of CO_2_ emitted from each treatment, excess amount of NaOH was back titrated against 1 M HCl^40^,^41^.

### Biochemical analysis

Soil samples were collected from each treatment after 90 and 180 days of incubation period for biochemical analysis. From each mesocosm, 100 g soil randomly sampled with the help of spatula and mixed in a sampling bottle. All samples were stored at 4 °C for further analysis. From composite sample, 5 g soil was taken in glass bottles and 12.5 mL of 0.1 M KCl was added. Subsequently, mineral N (NH_4_^+^-N, NO_3_^−^-N) content was extracted by continuous shaking of this mixture for 30 minutes on an automated reciprocal shaker^8^. After centrifuging this solution at 10000 rpm for 10 minutes, the supernatant was filtered through Whatmann filter paper (no. 42) and mineral N content was determined by Dionex ICS-5000+ DC (Thermo Scientific). FOSS Kjeltec™ 8400 Auto Sampler System (Eden Prairie, USA) measured the total N content in soil and grass litter. Electrical conductivity (EC) and pH was measured through multi-meter (Ino-Lab^®^ Multi 9430 IDS, WTW, GmbH & Co. KG, Germany) from a mixture of soil and distilled water (1:5, w-v ratio) which was shaken for 30 minutes. By using loss on ignition method as described for sandy soil^42^, organic matter and carbon content in soil and grass litter samples were estimated at a temperature of 850 °C.

Dissolved organic carbon in treatments at both sampling occasions was measured according to Ghani *et al*.^43^ after slight modification. In a 50 mL poly centrifuge tube, 3 g soil was weighed from each treatment, mixed with 30 mL distilled water and vortexed for 15 second. After tightly capping, poly tubes were placed at 80 °C in an oven for 16 h. To suspend the carbon in solution mixture, tubes were vortex again for 10–15 seconds after taking out from oven, and then centrifuged at 3500 rpm for 20 minutes. A 0.45 μm carbon free syringe filter was used to filter the resultant supernatant. The filtrate was run through total organic carbon (TOC) analyzer (TOC-V_CPH_; Schimadzu, Japan) to determine the TOC from these samples.

### Microbial biomass carbon, nitrogen and iron

Microbial biomass carbon, nitrogen (C_mic_ and N_mic_) and iron was determined through fumigation extraction method^44^,^45^. From composite sample, 10 g field moist soil was weighed and equally divided into two halves. The first half (5 g) was put in desiccator for 24 h at 25 °C to fumigate with ethanol free chloroform. Afterward the fumigants were removed by placing the sample in hot water bath for 1 hour. Both non-fumigated and fumigated soils were extracted with 20 mL K_2_SO_4_ (0.5 M) for carbon and nitrogen on an automated reciprocal shaker for 30 min. After filtering with Whatmann filter paper (No. 42), TOC and N_tot_ was analyzed through TOC Analyzer (TOC-V_CPH_; Shimadzu, Japan) and FOSS Kjeltec™ 8400 Auto Sampler System (Eden Prairie, USA), respectively. Following equation (1) was used to calculate C_mic_ or N_mic_.


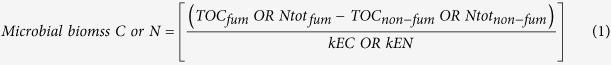


where kEC is 0.45, a constant^46^ used to calculate microbial biomass carbon and kEN is 0.54 for N_mic_ calculation^44^,^47^.

For iron, both fumigated and non-fumigated samples were extracted with 25 mL 1 M NH_4_NO_3_. After centrifugation, extract was acidified with suprapur HNO_3_ (Merck, Germany; 1:10 v/v) and stored at 4 °C. Labile concentration of iron in all samples was determined by inductively Coupled Plasma (ICPE-9000, Schimadzu, Japan) and calculated by subtraction of metal in non-fumigated sample from the metal in fumigated sample^48^.

Eco-physiological indicators were calculated to determine the nanoparticles pollution index on soil microbes and their associated functions. The ratio of respiration rate and C_mic_ is termed as metabolic quotient (qCO_2_)^49^. Moreover, microbial quotient (qM) is calculated as a ratio between microbial biomass and TOC^50^.

### Estimated nitrogen immobilization

Nitrogen immobilization was estimated by Kooijman *et al*.^39^, with some modification. Specifically, microbial efficiency (M_e_) was calculated from data of N:C ratios of leaf litter (NC_L_), microorganisms (NC_mic_), and respiration (R) and net nitrogen mineralization (N_min_) from litter applied treatments according to equation 2.


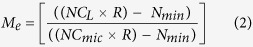


The calculated M_e_ was used to estimate nitrogen immobilization (N_im_) using following equation 3.


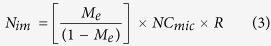


### Viable microbial cell counts

Pour plate method was used to estimate the colony counts of cultivable heterotrophic soil bacteria and fungi after 90 and 180 days of grass litter incubation. In 250 mL conical flasks, 10 g soil was weighed from aggregated samples of each mesocosm and then 90 mL sterile buffered peptone water (Merck, Darmstadt, Germany) was added to make a soil suspension. The suspension was shaken for 30 minutes on orbital shaker (140 rpm). Subsequently, we prepared serial dilutions from the soil suspended solution that were mixed with molten nutrient agar (HiMedia, USA) for bacteria as well as sabouraud dextrose agar (Himedia, USA) for fungi and poured in petri plates. These plates were incubated for 3 days at 30 ± 1 °C for bacteria and for 5 days at 25 ± 1 °C in case of fungi. All these experiments were carried out in triplicates. Afterwards, colony counter (ColonyCount V, Gerber Instruments AG, Effretikon, Switzerland) was used to record the colony forming units (cfu mL^−1^).

### Statistical analysis

To study the treatment, time and their interaction effects, univariate analysis of variance (ANOVA) was performed through SPSS 17.0 statistical package (IBM, New York, USA). The treatment effects were considered significantly different at 5% probability level. Time dependent effect of nanomaterial on various biochemical parameters was also tested using ANOVA at 5% probability. When main effects among treatments were significant the comparison analysis was performed by using Tukey test. Moreover, relationship of various biochemical parameters among each other and with carbon and nitrogen mineralization were tested for PCA on correlation matrices using Canoco 5 for Windows (Microcomputer Power Inc., Ithaca, NY) after 90 and 180 days of incubation study.

## Additional Information

**How to cite this article:** Rashid, M. I. *et al*. Toxicity of iron oxide nanoparticles to grass litter decomposition in a sandy soil. *Sci. Rep.* **7**, 41965; doi: 10.1038/srep41965 (2017).

**Publisher's note:** Springer Nature remains neutral with regard to jurisdictional claims in published maps and institutional affiliations.

## Figures and Tables

**Table 1:** Mean of the (n = 3) soil chemical parameters, pH, electrical conductivity (EC), total nitrogen (TN), organic matter (OM), total organic carbon (TOC) as well as carbon and nitrogen mineralization efficiencies (CME and NME, respectively) after 90 and 180 days of incubation study in control soil (CS), grass litter-amended soil (LS) and nanoparticles, grass litter amended soil (LNPS).

Treatment (T)		pH	EC	TN	OM	TOC	^¶^CME	^∥^NME
			dS m^−1^	mg kg^−1^	%			
**90 days**								
CS		8.67 ± 0.02a	1.50 ± 0.01a	123.13 ± 42.24	0.29 ± 0.08	0.17 ± 0.05	**—**	**—**
LS		8.35 ± 0.05b	2.14 ± 0.04b	218.33 ± 3.18	0.69 ± 0.06	0.40 ± 0.04	31.54 ± 2.16b	9.03 ± 2.10b
LNPS		8.35 ± 0.04b	2.24 ± 0.03b	152.00 ± 18.50	0.89 ± 0.55	0.51 ± 0.32	−20.18 ± 1.42a	−5.58 ± 1.37a
**180 days**								
CS		8.86 ± 0.05a	1.47 ± 0.015a	117.00 ± 31.23	0.48 ± 0.44	0.28 ± 0.26	**—**	**—**
LS		8.46 ± 0.04b	2.07 ± 0.04b	223.60 ± 76.35	1.01 ± 0.02	0.59 ± 0.01	34.64 ± 2.49b	15.64 ± 1.06b
LNPS		8.43 ± 0.02b	2.07 ± 0.14b	161.08 ± 52.08	0.87 ± 0.02	0.51 ± 0.01	−26.96 ± 0.55a	**−**13.79 ± 4.29b
	df			**Statistics (F-value)**				
T	2	62.520	23.539	2.643	1.810	1.810	760.725	**83.741**
Time	1	13.183	0.255	0.008	0.477	0.477	0.814	**4.610**
T × Time	2	0.427	0.093	0.015	0.167	0.167	5.954	**6.770**
**Statistics (P-value)**								
T	2	**0.000**	**0.000**	0.112	0.206	0.206	**0.000**	**0.000**
Time	1	**0.003**	0.623	0.931	0.503	0.503	0.481	**0.053**
T × Time	2	0.662	0.912	0.985	0.848	0.848	**0.045**	**0.035**

Small letters indicate the significant differences among treatments in column per time interval at 5% probability level. ¶
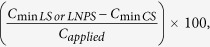
 
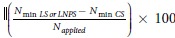
.

**Table 2:** Initial chemical characteristics such as dry matter (DM), total nitrogen (TN), organic matter (OM), mineral nitrogen (N_mineral_), organic nitrogen (N_organic_), carbon to nitrogen ration (C:N), pH and electrical conductivity (EC) of soil and grass litter.

	DM	OM	N_total_	N_mineral_	N_organic_	C:N ratio	pH (KCl)	EC
	(%)		g kg^−1^ DM					dS m^−1^
Soil	89.8 ± 0.07	0.29 ± 0.08	6.50 ± 0. 60	0.013 ± 0.01	6.49 ± 0.10	18.0 ± 1.31	8.7 ± 0.02	1.55 ± 0.02
Litter	—	95.30 ± 7.51	9.35 ± 1.35	0.005 ± 0.00	9.35 ± 1.35	21.4 ± 1.10	5.9 ± 0.12	—
